# Novel Compound Heterozygous Variants in *MKS1* Leading to Joubert Syndrome

**DOI:** 10.3389/fgene.2020.576235

**Published:** 2020-10-14

**Authors:** Minna Luo, Ruida He, Zaisheng Lin, Yue Shen, Guangyu Zhang, Zongfu Cao, Chao Lu, Dan Meng, Jing Zhang, Xu Ma, Muqing Cao

**Affiliations:** ^1^National Research Institute for Family Planning, Beijing, China; ^2^National Human Genetic Resources Center, Beijing, China; ^3^Key Laboratory of Cell Differentiation and Apoptosis of Chinese Ministry of Education, Department of Pathophysiology, Shanghai Jiao Tong University School of Medicine, Shanghai, China; ^4^Department of Children Rehabilitation, The Third Affiliated Hospital of Zhengzhou University, Zhengzhou, China; ^5^Tianjin Key Laboratory of Food and Biotechnology, School of Biotechnology and Food Science, Tianjin University of Commerce, Tianjin, China

**Keywords:** cilia, ciliopathy, Joubert syndrome, B9 proteins, *MKS1*

## Abstract

Joubert syndrome (JBTS) and Meckel–Gruber syndrome (MKS) are rare recessive disorders caused by defects of cilia, and they share overlapping clinical features and allelic loci. Mutations of *MKS1* contribute approximately 7% to all MKS cases and are found in some JBTS patients. Here, we describe a JBTS patient with two novel mutations of *MKS1*. Whole exome sequencing (WES) revealed c.191-1G > A and c.1058delG compound heterozygous variants. The patient presented with typical cerebellar vermis hypoplasia, hypotonia, and developmental delay, but without other renal/hepatic involvement or polydactyly. Functional studies showed that the c.1058delG mutation disrupts the B9 domain of MKS1, attenuates the interactions with B9D2, and impairs its ciliary localization at the transition zone (TZ), indicating that the B9 domain of MKS1 is essential for the integrity of the B9 protein complex and localization of MKS1 at the TZ. This work expands the mutation spectrum of *MKS1* and elucidates the clinical heterogeneity of *MKS1*-related ciliopathies.

## Introduction

Caused by defects of cilia, a variety of disorders are named ciliopathies (Hildebrandt et al., 2011; Novarino et al., 2011; Reiter and Leroux, 2017). Joubert syndrome (JBTS) and Meckel–Gruber syndrome (MKS) are two kinds of typical ciliopathies sharing overlapping clinical phenotypes, such as central nervous system malformation, renal/liver disease, and polydactyly (Brancati et al., 2010; Sattar and Gleeson, 2011; Hartill et al., 2017). JBTS is characterized by cerebellar vermis hypoplasia, which shows a typical molar tooth sign (MTS) on magnetic resonance imaging (MRI) (Joubert et al., 1969; Bachmann-Gagescu et al., 2020). Additional clinical symptoms may also be associated with brain malformations, including cystic kidney disease, liver fibrosis, polydactyly, or retinal dystrophy (Bachmann-Gagescu et al., 2020). Most JBTS patients are expected to have normal life spans (Dempsey et al., 2017). MKS is a rare lethal ciliopathy characterized by occipital encephalocele, cystic renal dysplasia, and postaxial polydactyly (Hartill et al., 2017). Ciliary defects in MKS result in perinatal lethality.

Genetic analysis has revealed that JBTS and MKS share more than 10 allelic loci (Kyttala et al., 2006; Baala et al., 2007; Valente et al., 2010; Dowdle et al., 2011; Garcia-Gonzalo et al., 2011), most of which encode proteins concentrated to the ciliary transition zone (TZ), a specialized region at the ciliary base controlling the composition of cilia (Chih et al., 2011; Garcia-Gonzalo et al., 2011; Sang et al., 2011). MKS1 is a TZ protein found in MKS, JBTS, and Bardet–Biedl syndrome (BBS), which is a ciliopathy characterized by obesity, retinitis pigmentosa, polydactyly, intellectual disability, and renal abnormalities (Kyttala et al., 2006; Dawe et al., 2007; Slaats et al., 2016). Mutations of the *MKS1* gene contribute to approximately 7% of all reported MKS cases (Hartill et al., 2017), but only a few mutations in *MKS1* have been reported to cause JBTS (Romani et al., 2014; Bachmann-Gagescu et al., 2015; Bader et al., 2016; Irfanullah et al., 2016; Vilboux et al., 2017). In the present study, we identified a patient with two novel *MKS1* mutations in a JBTS cohort. In contrast to most reported JBTS cases with ≥ 1 non-truncating mutation of *MKS1*, this patient carried two mutations leading to truncated forms of MKS1. Further studies showed that the truncated protein failed to interact with B9D2 and attenuated the TZ localization. These findings extend the spectrum of *MKS1* mutations in ciliopathies.

## Materials and Methods

### Whole Exome Sequencing and Variants Analysis

The exomes were captured by using the Agilent SureSelect Human All Exon V6 Kit (Agilent Technologies Inc.) and sequencing on an Illumina NovaSeq 6000 platform (Illumina Inc., CA, United States). Burrows−Wheeler Aligner and SAMtools were used to align the NGS reads to the hg19 reference genome (GRCh37). PCR duplicates were removed by Picard tools. Variants and small InDels were called by Genome Analysis Toolkit (GATK), annotated with Ensembl Variant Effect Predictor (McLaren et al., 2016) and filtered as described previously (Luo et al., 2019). Finally, all the variants were annotated according to American College of Medical Genetics and Genomics (ACMG) guidelines (Richards et al., 2015), and the variants from known causative genes of JBTS were analyzed with priority. Sanger sequencing was performed for variant validation.

### RNA Extraction and PCR

Tempus^TM^ Blood RNA Tube (Thermo Fisher Scientific, MA, United States) and Tempus^TM^ Spin RNA Isolation Kit (Thermo Fisher Scientific, MA, United States) were used for blood collection and RNA extraction, respectively. SuperScript IV First-Strand Synthesis System Kit (Thermo Fisher Scientific, MA, United States) was used for reverse transcription. The forward primer (5′-CCGAGTCCACCTGCAAAGAA-3′) used for cDNA amplification crossed the junction of exon 1 and exon 2, while the reverse primer (5′-TTCTCCCCCTCCGTCTCAAT-3′) was located at the beginning of exon 8. For qPCR, the primers of *MKS1* 5′-AAGGTGGCTCACTTCTCCTACC-3′ and 5′-AGAGGACCTCACAGTAGAGCAC-3′ and the primers of *GAPDH* 5′-GTCTCCTCTGACTTCAACAGCG-3′ and 5′-ACCACCCTGTTGCTGTAGCCAA-3′ were used.

### Plasmid Constructs

The cDNAs of *MKS1* and *B9D2* were amplified by PCR from a cDNA library prepared from HEK293 cells. *MKS1* variants were cloned into the pLV-FLAG lentiviral vector. *B9D2* was inserted into pCMV-HA. All plasmid sequences were validated by Sanger sequencing.

### Cell Culture, Plasmid Transfection, Lentivirus Package, and Infection

HEK293T cells were grown in Dulbecco’s modified Eagle medium (DMEM, Sigma, MO, United States) supplemented with 10% FBS (Sigma, MO, United States), 100 U/ml penicillin, and 0.1 mg/ml streptomycin. RPE1 cells were grown in DMEM/F12 (1:1 mixture) (Sigma, MO, United States) supplemented with 10% FBS, 100 U/ml penicillin, and 0.1 mg/ml streptomycin. Transfections of HEK293T cells were performed using linearized polyethyleneimine (PEI) (Polysciences Inc., PA, United States). pLV-FLAG plasmids harboring *MKS1* variants were transfected into HEK293 cells with psPAX2 and pMD2.g by the PEI method. Media containing virus were filtered through a 0.45 μm membrane filter and added into plates with RPE1 cells in the presence of 6 μg/ml polybrene (Santa Cruz, TX, United States). After 48 h of infection, RPE1 cells were selected with 10 μg/ml puromycin for 2 weeks.

### Co-immunoprecipitation and Western Blot Analysis

For co-immunoprecipitation (co-IP), transfected HEK293T cells were rinsed with ice-cold PBS and scraped into IP lysis buffer (20 mM Tris–HCl, pH = 7.5; 150 mM NaCl; 0.5 mM EDTA; and 0.5% Triton X-100) supplemented with protease inhibitor cocktail. After 20 min, cell lysates were cleared by 10,000 × *g* centrifugation at 4°C for 10 min. The supernatant was used for the co-IP assay by shaking with FLAG M2 beads (Sigma-Aldrich, MO, United States) for 2 h at 4°C. After three washes, proteins binding to FLAG beads were eluted with IP buffer containing 200 μg/ml 3 × FLAG peptides (Sigma-Aldrich, MO, United States).

For Western blot analysis, whole cell lysates or the elution products from the co-IP were denatured with 2 × SDS sample buffer, resolved on SDS-polyacrylamide gel electrophoresis, and subjected to Western blotting. The following antibodies were used for Western blot analysis: anti-FLAG (1:5,000, Sigma-Aldrich, MO, United States), anti-HA (1:3,000, Sigma-Aldrich, MO, United States), goat anti-mouse IRDye 680RD (1:15,000; LI-COR, NE, United States), and goat anti-rabbit IRDye 800CW (1:20,000; LI-COR, NE, United States).

### Immunostaining and Confocal Imaging

For immunostaining, RPE1 cells were seeded on coverslips in six-well plates. After serum starvation for 48 h, cells were washed with PBS and fixed with 4% paraformaldehyde for 10 min, and the fixed cells were permeabilized by −20°C cold methanol or 0.3% Triton X-100 for 10 min. After washing twice with PBS, cells were stained using primary antibodies in blocking buffer (PBS containing 1% bovine serum albumin and 0.1% Triton X-100) for 1 h at room temperature. After washing with PBS twice, cells were incubated with secondary antibodies in blocking buffer for 1 h at room temperature. DNA was visualized by DAPI (Sigma-Aldrich, MO, United States). Samples were visualized using a FV3000 confocal microscope (Olympus, Tokyo, Japan) with a 40 × /NA1.4 objective (Olympus, Tokyo, Japan). Data from three independent experiments was used for intensity quantification.

## Results

### Novel Mutations of *MKS1* Are Associated With Joubert Syndrome

**FIGURE 1:**
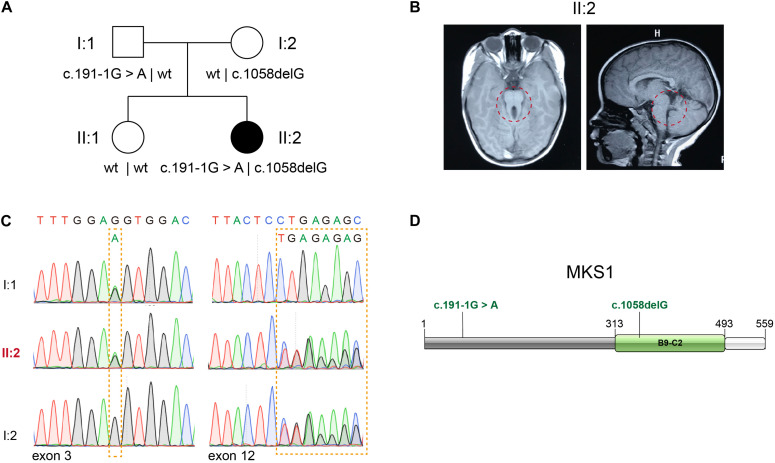
Identification of novel *MKS1* mutations in a patient with Joubert syndrome. **(A)** Pedigree of family referred for a clinical diagnosis of Joubert syndrome (JBTS). **(B)** Brain MRI of the proband showing the typical molar tooth sign and cerebellar vermis hypoplasia. **(C)** Electropherograms of Sanger sequences showing *MKS1* sequences of the proband and her parents. **(D)** Schematic diagram of MKS1 domain structure and the mutations of the affected individual. The B9 domain is labeled as B9-C2.

Whole exome sequencing (WES) of 151 patients in a JBTS cohort resulted in the identification of two likely pathogenic *MKS1* variants. The proband, II:2, is the second child of non-consanguineous parents without related medical history (Figure 1A). She was born full-term by normal delivery, weighing 4,050 g and measuring 55 cm in height. The proband was hospitalized at the age of 5 months, and she was diagnosed with hypotonia and developmental delay. Brain MRI showed typical molar tooth sign, indicating severe cerebellar vermis hypoplasia (Figure 1B). No renal/hepatic involvement, polydactyly, or agenesis of the corpus callosum was observed (Table 1). Several rare deleterious variants in the known JBTS genes were found by WES (Supplementary Table 1). WES found the compound heterozygous variants in the *MKS1* gene (GenBank: NM_017777.3), but other variants are heterozygous (Supplementary Table 1). The paternally inherited variant of *MKS1* is mutated at the canonical splice acceptor site, and it is also known as rs201362733 with a rare frequency (0.00001202) in gnomAD (Figures 1C,D). The maternally inherited variant, c.1058delG, is a deletion of one nucleotide causing a frameshift and premature stop, p.G353Efs^∗^2 (Figures 1C,D). Thus, both of the two variants of *MKS1* were classified as “pathogenic,” according to the ACMG guidelines (Richards et al., 2015). The absence of these two variants in her sister revealed that she is not a carrier.

**TABLE 1:** Clinical features and genotype.

Sample name	129C
Gender	Female
Age	5 months
Ethnic	Chinese
Mutation 1	c.191-1G > A
	p.S64Mfs*12
Mutation 2	c.1058delG
	p.G353Efs*2
Molar tooth sign	+
Developmental delay	+
Respiratory abnormality	–
Hypotonia	+
Oculomotor apraxia	–
Retinal involvement	NA
Renal involvement	–
Liver involvement	–
Limb anomalies	–
Agenesis of the corpus callosum	–

### Confirmation for an Abnormal Transcript of *MKS1*

**FIGURE 2:**
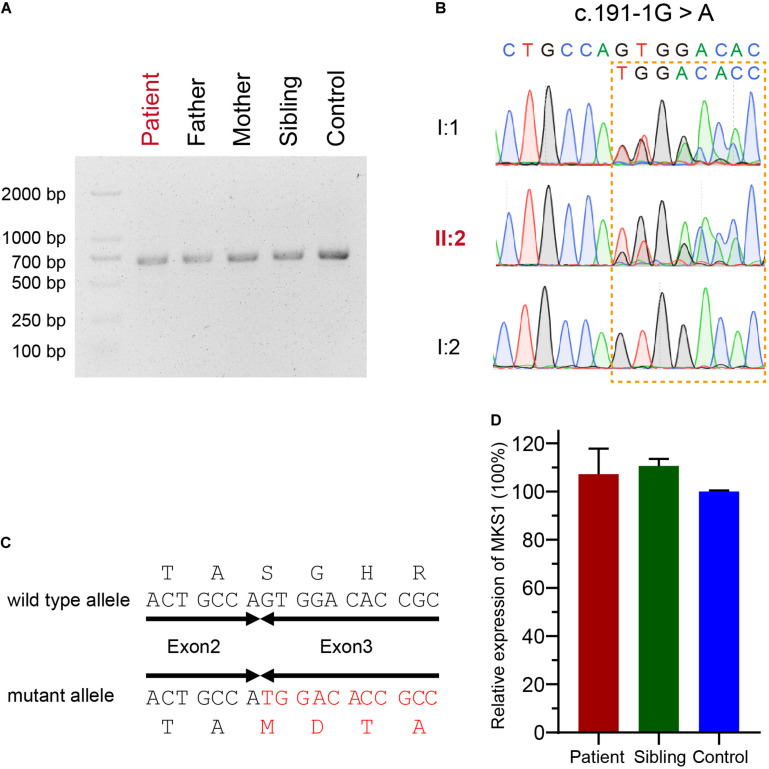
Confirmation for an abnormal transcript of *MKS1*. **(A)** Image of agarose gel electrophoresis of the PCR products from the proband, her parents, and sibling and the healthy control. **(B)** Chromatograms showing the DNA sequences of wild type and c.191-1G > A mutation. **(C)** The predicted protein sequences translated from mRNAs of wild type and c.191-1G > A mutation. **(D)** Relative MKS1 mRNA levels of the patient, the sibling, and a health control. Data from three independent experiments was used for the quantification. Error bars represent the SD.

The mutational effect of c.191-1G > A was validated by reverse transcription PCR. Surprisingly, only one band was observed in the proband, her father, her mother, her sister, and a healthy control (Figure 2A). Sanger sequencing showed one nucleotide deletion at the beginning of exon 3 rather than skipping exon 3, which was caused by the failure of correct splicing in c.191-1G > A mutated samples (the proband and father) (Figures 2B,C). This deletion resulted in a frameshift and a premature stop, which is annotated as p.S64Mfs^∗^12. The mRNA level was comparable to the sibling and healthy control (Figure 2D).

### The B9 Domain of MKS1 Is Required for the Transition Zone Localization and Interaction With B9D2

**FIGURE 3:**
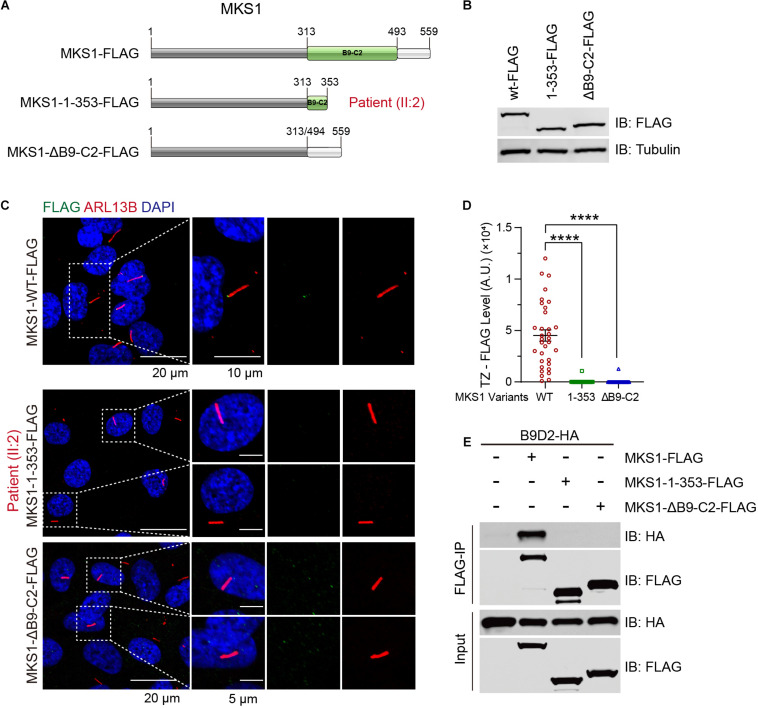
MKS1 mutant fails to localize at the transition zone and lose the interaction with B9D2. **(A)** Schematic diagram of the domain structure of MKS1 variants (wild type, MKS1 1-353, and MKS1 lacking B9 domain). **(B)** Immunoblot analysis of RPE1 cell lines stably expressed FLAG-tagged MKS1 variants shown in **(A)**. **(C)** Immunostaining of RPE1 cells expressing FLAG-tagged MKS1 variants. MKS1 variants (FLAG, green), cilia (ARL13B, red), and nuclei (DAPI, blue). **(D)** Quantification of MKS1 variant levels at the transition zone. Each dot represents the signal of one cell. Data from three independent experiments was used for the quantification. Error bars represent mean ± SD. Statistical significance was determined by an unpaired Student’s *t*-test (****p < 0.0001). **(E)** Immunoprecipitation of FLAG-tagged MKS1 variants with B9D2.

B9-containing proteins are highly conserved proteins present only in organisms assembling cilia (Barker et al., 2014). *MKS1* encodes a protein with 559 amino acids and the B9 domain localizes at the C-terminus. The c.191-1G > A variant was predicted to generate a short peptide containing 64 correct amino acids, suggesting complete loss of function. The c.1058delG variant caused early termination of the translation of MKS1, which caused the partial loss of the B9 domain (314–493) and loss of the C-terminal tail (494–559). To confirm the pathogenicity, we established RPE1 cell lines stably expressing FLAG-tagged wild-type, MKS1 1-353 (to mimic p.G353Efs^∗^2), or B9 domain lacking MKS1 (Figure 3A). Immunoblots confirmed the expression of the variants (Figure 3B). Previous studies have shown that MKS1 mainly localizes at the ciliary transition zone (Dowdle et al., 2011; Garcia-Gonzalo et al., 2011; Sang et al., 2011; Slaats et al., 2016). Therefore, we determined whether the MKS1 1-353 disrupted the localization of MKS1 by immunostaining. Almost all cells expressing wild-type *MKS1* had FLAG signal at the transition zone, but the localization of MKS1 1-353 or the B9 domain-lacking mutant was largely attenuated (Figures 3C,D). It has been shown that the three B9 proteins, MKS1, B9D1, and B9D2, form a protein complex, the integrity of which is essential for their function (Chih et al., 2011; Dowdle et al., 2011; Garcia-Gonzalo et al., 2011; Sang et al., 2011). In this complex, MKS1 predominantly interacts with B9D2 (Dowdle et al., 2011). To test whether the mutation affects B9 protein complex formation, we performed co-immunoprecipitation assays. Immunoblots showed that wild-type MKS1 but not the MKS1 1-353 or B9 domain-lacking MKS1 interacted with B9D2 (Figure 3E). These results demonstrate that the B9 domain is essential for the localization and activity of MKS1 protein.

## Discussion

In this study, we report a JBTS patient with two novel *MKS1* mutations displaying global developmental delay, MTS, and hypotonia. Additional brain anomalies such as agenesis of the corpus callosum, which is more frequent in MKS than JBTS (Bader et al., 2016), were not observed. This patient belongs to JBTS, because no renal or liver involvement and no limb anomalies were displayed.

A hypothesis has been proposed for the genotype–phenotype correlation as follows: two null alleles of *MKS1* result in MKS; one null allele and one hypomorphic allele result in JBTS; and two hypomorphic alleles result in BBS (Bader et al., 2016). In this study, the proband has two truncating mutations but has JBTS. This situation has also occurred in three other cases as follows: homozygous splice acceptor site mutation of c.1461-2A > G in COR340 (Romani et al., 2014), homozygous frameshift mutation of c.1528dupC (p.R510Pfs^∗^81) in UW31-3, and homozygous splice acceptor site mutation of c.1589-2A > T in UW150-3 (Bachmann-Gagescu et al., 2015; Slaats et al., 2016). Taken together, these findings suggested that the genotype–phenotype correlation is more complicated in *MKS1*-mutated ciliopathies.

Previous landmark studies have shown that the integrity of the B9 complex may be essential for the control of the entry and exit of ciliary components (Dowdle et al., 2011; Sang et al., 2011). We found that loss of the B9 domain of MKS1 largely attenuates its transition zone localization and disrupts the interaction with B9D2. These findings are consistent with previous studies. Three major protein modules locate at the TZ region, namely the NPHP complex, the B9 complex, and the TMEM–TCTN complex. The disruption of the B9 complex possibly changes the structure of the three key modules and results in ciliopathies.

In summary, we identified two novel null mutants of *MKS1* resulting in JBTS, expanding the genetic basis of JBTS. Our findings further implicate that clinical features of *MKS1* mutations are more complicated than the previously proposed genotype–phenotype correlation model of the *MKS1* gene. Finally, these findings will be helpful for the genetic testing of JBTS patients and their families.

## Data Availability Statement

The data in this study are available from MC, upon reasonable request.

## Ethics Statement

The studies involving human participants were reviewed and approved by the Ethics Committee of the National Research Institute for Family Planning of China. Written informed consent to participate in this study was provided by the participants’ legal guardian/next of kin. Written informed consent was obtained from the individual(s), and minor(s)’ legal guardian/next of kin, for the publication of any potentially identifiable images or data included in this article.

## Author Contributions

MC, XM, JZ, and ML conceived and directed the project. GZ collected the blood sample and medical information of the patients. YS analyzed and interpreted the medical data. ML, YS, and CL prepared the samples and performed the WES. ZC and ML analyzed and interpreted the WES data. RH, ZL, and DM performed the biochemical analysis and imaging. MC, XM, JZ, and ML wrote the manuscript with the help of all the other authors. All authors contributed to the article and approved the submitted version.

## Conflict of Interest

The authors declare that the research was conducted in the absence of any commercial or financial relationships that could be construed as a potential conflict of interest.
